# Iranian children with overweight and obesity: an internet-based interventional study

**DOI:** 10.1186/s12887-021-02684-2

**Published:** 2021-05-06

**Authors:** Farnaz Khatami, Ghazal Shariatpanahi, Hamid Barahimi, Rezvan Hashemi, Leila Khedmat, Mahta Gheirati

**Affiliations:** 1 Department of Community Medicine, School of Medicine, Tehran University of Medical Sciences, Tehran, Iran; 2 Department of Family Medicine, Ziaeian Hospital, Tehran University of Medical Sciences, Tehran, Iran; 3 Department of Pediatrics, Ziaeian Hospital, Tehran University of Medical Sciences, Tehran, Iran; 4 Department of Geriatric Medicine, Ziaeian Hospital, School of Medicine, Tehran University of Medical Science, Tehran, Iran; 5 Health Management Research Center, Baqiyatallah University of Medical Sciences, Tehran, Iran

**Keywords:** Childhood obesity, Overweight, Practical education, Lifestyle modification, Nutritional pattern, Gender difference

## Abstract

**Background:**

Obesity or overweight in children is an excessive accumulation of adipose tissue that can potentially regress health indicators and increase the likelihood of various diseases.

**Objectives:**

This model was implemented to improve the nutritional status and lifestyle behavior of children aged 6–12 years with overweight/obesity.

**Methods:**

A quasi-experimental design with 90 participants in each control and intervention group with a multistage cluster random sampling method after reviewing the literature, and their screening by experts were adopted.

**Results:**

After 6 months there were significant differences in Body Mass Index and weight for age percentile values of children allocated in control and intervention groups after controlling for beginning values (*p* = 0.024, Partial eta2 = 0.028, 0.044, Partial eta2 = 0.023), respectively. Although there was an increased rate in BMI and weight for age percentile in both groups this increase in the control group after the 6th month significantly was more than that in the intervention group after the 6th months. A considerable difference in BMI of girls after the intervention was observed in the experimental group (*p* = 0.006, Partial eta2 = 0.092). However, our results showed that there was no significant difference in BMI of boys in the intervention and control groups before and 6 months after the intervention (*p* = 0.507).

**Conclusions:**

We conclude that though the weight increase rate was lower in the experimental group, the implemented model alone was not enough.

**Trial registration:**

Iranian Registry of Clinical Trials (IRCT): IRCT20200717048124N1 at 05/08/2020, retrospectively registered.

## Introduction

Obesity or overweight is excessive accumulation of adipose tissue that can potentially regress health indicators, such as reducing average life expectancy or quality [1]. These emerging health issues increase the likelihood of various diseases, especially heart diseases, type 2 diabetes, sleep apnea blockers, certain types of cancer [2, 3]. Overweight and obesity are mostly caused by a combination of excessive energy intake, low physical activity, and genetic predisposition. However, few cases are initially caused by genes, endocrine disruptions, medications, or mental illness [4, 5]. Diet and exercise are the main solutions to reduce overweight and obesity [6]. Over the past two decades, the prevalence rate of obesity is 7–16% in children and adolescents of primary school age. Iran is one of seven countries with the highest incidence of childhood obesity in the world. Studies showed that the prevalence rate of obesity and overweight, as well as abdominal obesity among 14-year-old boys in Tabriz city (north-west of Iran), was 20 and 16%, respectively [7, 8]. Increased intake of high-fat foods, decreased physical activity, and cultural factors such as knowledge, attitudes, and nutritional behaviors may play an important role in causing childhood obesity [9]. Prevention of these health problems is of great importance due to the numerous medical and psychosocial consequences that emerged in this age group. Most obesity-related studies conducted in Iran highlighted the inadequate status of the components of children’s and adolescents’ lifestyles [10, 11]. Although different methods have been suggested to treat childhood obesity, the best treatment always is prevention through the training 3 of proper lifestyles and the implementation of behavior change programs. Research has also shown that educational programs are useful not only in treating obesity but also in reducing stress and improving the sense of adequacy and body image [12]. On the other hand, school programs have many benefits for implementing childhood obesity prevention programs, including the high availability to a large number of children, the continuity in educational guidance, minimal costs, and the comfortable participation of parents in the programs [9, 11]. Meanwhile, the Internet, as a cost-effective and the ideal way can also be utilized to provide a wide range of lifestyle interventions [13]. There is a necessity to reduce or prevent childhood overweight and obesity through shaping a healthy lifestyle program due to the high potential of educational environments to train children, as well as the lack of a practical web-based educational model for family physicians. Therefore, this study was aimed to determine the effectiveness of an interventional model to modify nutritional status and lifestyle of children aged 6–12 years by reducing Body Mass Index (BMI) and weight for age values.

## Methods

### Study design and population

An interventional study was designed in the primary schools for girls and boys located in the 17th district of Tehran for 6 months from April 2019 to November 2019. A multistage sampling process was used to select schools. Among girls’ schools, 2 schools were selected as the intervention group (first and third choice) and 2 schools (second and fourth choices) as the control group. Also, among boys’ schools, 2 schools were selected as the intervention group (first and third choices) and 2 schools (second and fourth choices) as the control group. Then, in each school, eligible students were determined in clusters at a grade level and everyone within the chosen clusters entered the control or intervention group using the sealed opaque envelopes. One hundred and eighty children aged 6–12 years with overweight and obesity were allocated to two groups of intervention (*n* = 90) and control (*n* = 90). The sample size (n) with 5% error rate and 80% statistical power was assessed using the following equation (Eq. 1) [14, 15]:

1 $$ n=\frac{{\left({Z}_1-\frac{\alpha }{2}\right)}^2\left({\sigma}_1^2+{\sigma}_2^2\right)}{{\left({\mu}_2-{\mu}_1\right)}^2} $$

Only the statistical consultant in this study was blinded.

### Inclusion and exclusion criteria

Students with obesity and overweight assessed by the Centers for Disease Control and Prevention (CDC) growth charts were qualified for participation in this project. Based on the Body Mass Index (BMI) reported by the CDC growth charts, overweight (85th percentile ≤ BMI < 95th percentile), and obese (95th percentile ≤ BMI) children in this research were considered according to the ender and age range [16]. However, children with underweight and normal weight at the same age range, having the underlying illness, the lack of access to the Internet and the lack of willingness to cooperate were excluded.

### Intervention program: design and implementation

The analysis, design, development, implementation, and evaluation (ADDIE) development model study was used to assess the best tool to educate schoolchildren about healthy eating and physical activity to reduce their obesity and overweight. The ADDIE, as 5 a systematic instructional design model, empowers training professionals and designers to develop more principled and effective training through a dynamic and flexible guideline. This approach is based on all theories of cognition, behaviorism, and constructivism. This model covers the design of learning topics and perspectives and different environments and is therefore called a generic paradigm [17]. This model applies to any learning, both traditional and electronic. The ADDIE model’s phases include (i) analysis, (ii) design, (iii) development, (iv) implementation, and (v) evaluation. Five steps must be taken for the design of the training so that each step is considered as an input for its next step [18]. Accordingly, the different steps were implemented in the present study as follows:

#### Phase I: analysis

An extensive literature review was initially performed to find a variety of educational models. It was decided to make the final decision after interviewing with experts and summarizing the opinions. Before the meeting, the purposes and the different issues were discussed concerning the selection of the weight loss methods and related articles. Some information was also collected on overweight and obese students, their educational needs, purposes, and practices, and study limitations.

#### Phase II: design

The model design was completed in a separate meeting with the relevant experts. According to the age group of children with overweight and obesity, education to their parents was approved using the website as an efficient, modern, and accessible method. The meeting also addressed the educational needs of students that had been previously collected. Fifteen expert panel members 6 including intervention researchers, healthcare professionals, and school practitioners selected the most relevant lifestyle modification interventions to reduce obesity and overweight risk factors. After giving the preliminary explanations and discussing the types of interventions at the meeting, two healthy lifestyle views were finally endorsed to be used on the educational site. These interventional patterns included (i) 5–2–1-0 healthy habit (HH), and (ii) healthy eating plate (HEP). The HH pattern is based on the daily consumption of five units or more fruits and vegetables, 2 h or less of everyday use of television or other electronic equipment, 1 h or more daily physical activity, and no sugary drinks [19]. The HEP is a guide to choosing a healthy and balanced diet. The main message of the HEP is to focus on the quality of the consumed food, which summarizes: (i) always try to make half of your plate full of fruits and vegetables, (ii) fill a quarter of the plate with whole grains, (iii) fill a quarter of the plate with protein sources, (iv) consume a balanced amount of vegetable oils, (v) drink water, tea or coffee, and (vi) do not forget about exercise [20]. It was decided to upload educational material on the “Peak-e-Salamat” website entitled “healthy living pattern for children aged 6-12 years old”. The “Peak-e-Salamat” is a website representing a categorized collection of web pages, images, multimedia files, or other digital tools related to the general and particular health issues that reside on one or more hosts known as web servers. “Peak e-Salamat” is also considered as an educational aid content generation system about understanding illnesses and self-care and provided information to the general population, patients, and medical professionals.

#### Phase III: development

Each of the two health patterns (5–2–1-0 HH and HEP) included training frameworks. In the form of instructional information, they were written in a simple language to put the content on the training website during the implementation phase.

#### Phase IV: implementation

After holding numerous meetings with educational institutions for justifying the research project, the necessary permits to start its implementation were obtained. The research team selected eligible children with overweight or obesity at elementary schools, during 30 days before the start of the study. After stating the mission of the study, and receiving a written informed consent from the parents, children who also had verbal agreement were included in the study. The importance of weight loss and its risks was emphasized in the first virtual session for the interventional group. Then the 5–2–1-0 HH and HEP patterns in reducing the weight accompanied by encouraging ways to the participation of students were educated. At the end of the sessions, some questions were designed to raise parental attention to continue the training. Educational materials were uploaded to the website twice a week. Parents were surveyed by forming a virtual group for questions and answers as well as sending personal text messages following the uploading of educational materials. Phone calls were also made once a month to answer their questions or concerns about the site or plan. Parental assessment method during the study approved by 15 experts. At the end of the 3rd month, the height and weight of the children were measured only in the intervention group. This process was repeated at the end of the 6th month in both groups. The expected outcome of this study was a further reduction in BMI and weight for age percentile and a comparative study at the beginning and end of the study. Participants were not exposed to greater or additional risks due to the lifestyle training intervention. There was no specific intervention for the control group but it included all the preventive recommendations and training that were part of the schools’ routine.

#### Phase V: evaluation

At this stage, the height and weight status data as well as children’s demographic data were entered into SPSS software package version 25.0 (SPSS Inc., Chicago, IL, USA) to analyze statistically, and then the report of results was written.

### Data collection

The demographic data, including student’s age and gender, parents’ age, student’s weight and height, family size, and parent’s educational level and occupational status, were collected through a self-designed questionnaire. The validity of the demographic questionnaire was confirmed by 15 experts. The height was determined without shoes in a standing position by a stadiometer to the nearest 0.5 cm, whereas the shoulders were in a normal state. The bodyweight of subjects wearing light clothes and no shoes or socks was measured and recorded using a standard, calibrated electronic scale (Xiaomi, China) with an accuracy of 100 g. The BMI as the primary outcome was determined by calculating the weight (in Kg) divided by the square of assessed height (in m^2^). Children Growth Chart Calculator of Center for Disease Control and Prevention (CDC) used to calculate the weight for age percentile as a secondary outcome which complements the results of the body mass index. The weight, height, BMI, and weight for age percentile were assessed before and after the six-month educational intervention.

### Data analysis

The data of categorical variables were expressed as frequency and percentage, whereas the results of numerical variables were represented as mean ± standard deviation. Inferential statistics in terms of independent and paired t-tests, Chi-squared (χ2) test/Fisher’s exact test, repeated-measures ANOVA, was carried out to compare the significant difference between means of different groups. The AVCOVA test used to analyze the difference of 6 months BMI/weight for age percentile, with beginning BMI/weight for age percentile as covariates. The SPSS software was used to analyze the data at a significant level of *p* < 0.05.

## Results

A meeting in the presence of community medicine specialists, pediatricians, nutritionists, and internists were held to determine the best model pattern and design for weight loss in children aged 6–12 years. The results of the literature review on the types of interventions to reduce childhood obesity and overweight were extracted and summarized in Table 1. After evaluating the different interventions by experts, two healthy lifestyle patterns (5–2–1-0 HH and HEP) were approved to be used in the intervention group through training by the “Peak-e-Salamat” website. Table 2 shows the demographic data of children groups. No significant difference in the gender between the two groups was observed (*p* = 0.454). The mean age of the participant in each group was the same (*p* = 0.956). Moreover, there was no significant difference in the average age of fathers and mothers in the two groups. The families of three and four persons were the most common family size in both groups. All subjects were present in both groups until the end of 6 months and no one was excluded from the study.

**Table 1:** A summary of the best lifestyle modification interventions to reduce obesity or overweight

Source	Methods	Main outcomes
Rogers et al., 2013 [21]	5–2–1-0 HH: The daily consumption of five units or more fruits and vegetables – Two hours or less everyday use of television or other electronic equipment - One hour or more daily physical activity - No sugary drinks	A significant increase in the intake of fruits and vegetables in children from 63 to 69% - The substantial reduction of sugars intake in children from 10 to 47%, both of which were significant - The notable increase of parental awareness
Kelishadi et al., 2009 [22]	Similar nutrition and behavioral therapy in all the study groups (A-C) -Attending in physical activity training courses, twice a week (group A) Providing educational CD (group B), −Face to face education (group C)	A remarkable reduction in BMI of participant allocated in groups A and B compared to the pre-intervention - No significant the difference in BMI value among the different groups
Mohammadi et al., 2013 [23]	An educational intervention including four 3- min group discussion sessions with photo presentation in groups of 15 people	Significant improvement in behaviors and self-efficacy in overweight and obesity-related lifestyle among students
Wang et al., 2015 [24]	Participants were divided into three groups: control, diet and exercise, physical activity alone, and diet alone.	The maximum decrease in BMI, waist circumference, and fat percentage with the integrated intervention of diet and exercise
James et al., 2007 [25]	Three educational sessions during the academic year based on healthy nutrition and encouragement not to use sugary drinks	An increase in mean BMI in experimental and control groups after a 2-y follow-up
Kim et al., 2017 [26]	Holding the meeting with the presence of experts and discussing various methods	The best method: controlling the diet and improving the physical activity, and b. The best scale to measure the weight gain: age and gender-based BMI percentile rank
Mâsse et al., 2014 [27]	Eight-month web-based electronic health intervention	Motivation was a key predictor factor for adolescents’ adherence to web- based intervention so that the entrance to the system decreased to 33.3% in the last 4 months of the intervention
Baranowski et al., 2003 [28]	A training program in a daily camp for 4 weeks and a subsequent online training intervention	The mean BMI: An insignificant increase of the case subjects along with a marginal decrease in control people after the 12 week-intervention

*HH* Healthy habit, *BMI* Body mass index

**Table 2:** The demographic characteristics of Iranian children in the control and intervention groups

	Group		*P*-value
	Control (*n* = 90)	Intervention (*n* = 90)	
	N (%)	N (%)	
Gender			
Male	47 (52.2)	52 (57.8)	0.454
Female	43 (47.8)	38 (42.2)	
Family size (n)			
3	32 (35.6)	29 (32.2)	0.925
4	39 (43.3)	44 (48.9)	
≥4	19 (21.1)	17 (18.9)	
Father’s educational level			
Till diploma	80 (88.9)	78 (86.7)	0.459
Academic	10 (11.1)	12 (13.3)	
Mother’s educational level			
Till diploma	89 (98.9)	85 (94.4)	0.211
Academic	1 (1.1)	5 (5.6)	
Father’s job			
Employee	16 (17.8)	18 (20)	0.703
Others	74 (82.2)	72 (80)	
Mother’s job			
Housewife	86 (95.6)	85 (94.5)	0.500
Others	4 (4.4)	5 (5.5)	
	Mean ± SD	Mean ± SD	
Student’s age (years)	10.8 ± 1.4	10.8 ± 1.4	0.956
Father’s age (years)	41.9 ± 4.27	41.3 ± 4.7	0.391
Mother’s age (years)	36.8 ± 5.1	36.1 ± 5.0	0.380

*SD* Standard deviation

**Fig. 1:**
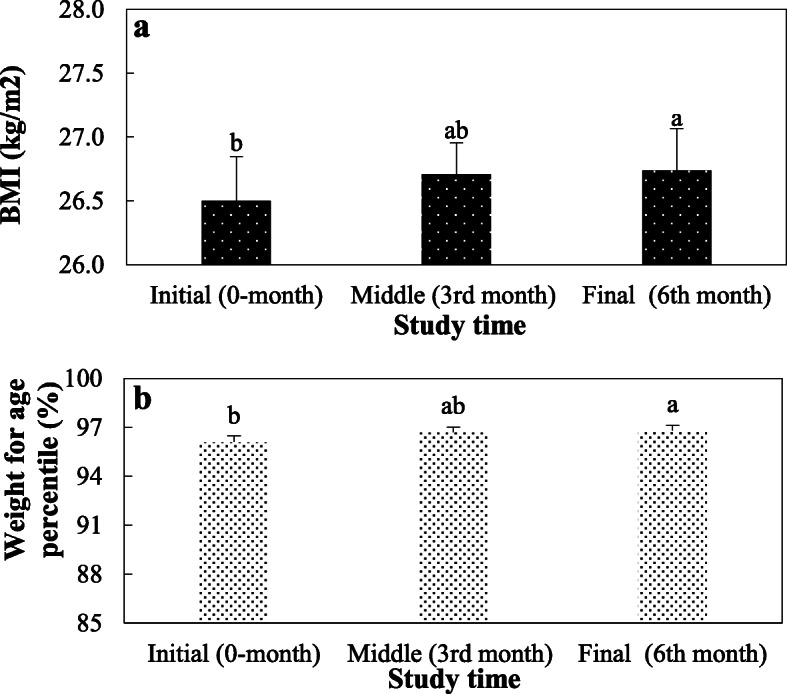
The Body Mass Index (BMI) (a) and weight for age percentile (b) of children in the intervention group in the 0, 3rd and 6th month of the study

Table 3 exhibits that there was no significant difference in BMI values of intervened and control children at the beginning of the study (*p* = 0.40). Moreover, the BMI of children allocated in the control and intervention groups at the 6th month was statistically different after controlled for BMI at the beginning (*p* = 0.024, Partial eta2 = 0.028). Weight for age percentiles of children in the two groups at the study beginning was determined to be 96.1 ± 3.6 and 95.7 ± 3.8, respectively (*p* = 0.50) and the impact of the intervention on the difference of percentile 0 and 6 months in groups was statistically significant (*p* = 0.044, Partial eta2 = 0.023) (Table 3). The repeated-measures ANOVA test (Fig. 1a) showed that there was a significant difference in BMI values of case children at the 0, 3rd, and 6th month of intervention (*p* < 0.001). This statistic examination also explored a significant increase in weight for age percentile for the case children at the 0, 3rd, and 6th month, (*p* < 0.001) (Fig. 1b). Although this parameter was significantly increased by prolonging the intervention time in each group, the increase in BMI in the intervention group was significantly lower than the control group. A considerable difference in BMI of girls after the intervention was observed in the experimental group (*p* = 0.006, Partial eta2 = 0.092). However, our results showed that there was no significant difference in the BMI of boys in the intervention and control groups before and 6 months after the intervention (*p* = 0.507). Also in weight for age percentile, the difference for girls after the intervention was significant (*p* = 0.018, Partial eta2 = 0.07). Also in intervention group, the main effect of sex on average BMI score and weight for age percentile was significant (F (1, 60) = 7.583, *p* = 0.008), (F (1, 60) = 5.702, *p* = 0.020), respectively.

**Table 3:** The effect of the program in control and intervention groups

	Control group	Intervention group	*P*-value
	(*n* = 90)	(*n* = 90)	
	Mean (SD)	Mean (SD)	
BMI:			
Beginning	26.3 ± 3.1 kg/m^2^	26.5 ± 3.3 kg/m^2^	0.40
After 3 month	–	26.71 ± 3.3 kg/m^2^	
After 6 month	26.5 ± 3.3 kg/m^2^	26.74 ± 3.1 kg/m^2^	0.024, Partial eta^2^ = 0.028
Weight for age percentile:			
Beginning	95.7 ± 3.8	96.1 ± 3.6	0.05
After 3 month		96.7 ± 3.0	
After 6 month	96.9 ± 2.7	96.8 ± 3.1	0.044, Partial eta^2^ = 0.023

*BMI* Body mass index, *SD* Standard deviation

## Discussion

The present study showed that there was no significant difference in BMI between the intervention and control girls before the intervention. However, the weight gain was significantly lower in the experimental group with Internet-based intervention. It was well demonstrated that lifestyle modification by limiting calorie intake to control weight is one of the first appropriate ways to prevent or treat obesity and overweight [29–31]. In our study, all groups gained weight, but this increase was significantly lower in the intervention group. James et al. after a two-year follow-up from the Christchurch obesity prevention program in schools, found that the BMI in the control and intervention groups was insignificantly increased by 2.14 and 1.88 units, respectively [25]. In a pilot study by Baranowski et al., it was earlier explored that the mean BMI in the case and control groups was insignificantly increased and decreased after the 12 week-intervention, respectively [28]. This discrepancy may be attributed to the difference in the intervention type and duration, as well as children’s age.

In general, diverse individual factors such as demographic variables, attitudes, personal beliefs, and environmental and social factors influence dietary compliance. Perceived benefits and obstacles to follow a regular nutritional program (RNP) are two positive and negative cognitive factors. The perceived barriers related to implementing an RNP among various demographic groups have been examined. These obstacles may be internal (such as negative attitudes) or interpersonal [32–36]. To achieve the goal of losing weight in obese or overweight children and adolescents, it is necessary to identify a set of the most important and effective individual, environmental and social factors associated with a healthy diet to design interventions and overcome obstacles and problems with diet therapy [37, 38]. Since the main purpose of the current study was to determine the impact of education on lifestyle modifications, the identified general and major needs were more focused on the second prevention of overweight and obesity with healthy diets. For this reason, educational intervention could not improve the part of lifestyle behaviors associated with physical activities. However, recommendations, guidelines, and comprehensive programs to increase levels of physical activity in schools have been provided by the CDC [39, 40].

Compared to some of the previous studies, the findings of the present investigation showed that the significant impact of education on the improvement of students’ weight-related lifestyles was different [22–24]. Mohammadi Zeidi et al. reported that the educational intervention could remarkably improve the behavior and self-efficacy affecting overweight and obesity-related lifestyle in students [23]. Wang et al. found that the combined intervention program based on diet and exercise guidelines had the highest decrease in BMI, waist circumference, and fat percentage compared to the control, and each group trained with dietary and physical activity interventions alone [24]. Kelishadi et al. earlier mentioned that the BMI of individuals who were participated in physical activity classes or trained with CD education was significantly decreased compared to pre-intervention [22].

In our study, there was no significant difference between the mean BMI of children and parents’ age in the intervention and control groups, and age did not affect their weight gain or decrease. Similar results were reported by Mâsse et al. after the implementation of a web-based intervention [27]. Mohammadi Zeidi et al. also showed that the mean age of children and their parents did not affect child weight change [23]. Moreover, Wang et al. reported that the mean age of the dietary intervention group had no significant effect on weight change [24].

There was no significant difference in the BMI of girls allocated between the intervention and control groups before the intervention. Nevertheless, a considerable decrease in BMI of girls after the intervention was observed in the experimental group. However, our results showed that there was no significant difference in the BMI of boys in the intervention and control groups before and 3 months after the intervention. Overall, a significant difference between the two groups before and after the intervention was found. The findings of this study were consistent with previous studies regarding the intervention effectiveness in girls and lack of effectiveness in boys. The influence of peers on the transition from childhood to adolescence plays an important role in improving girls’ physical activity and reducing their BMI [41, 42]. Sebire et al. have recently reported the effectiveness of the peer-led school-based intervention in promoting physical activity in adolescent girls [43]. Therefore, the observed differences in the effect of peer education on girls’ peers and their ineffectiveness in boys may be due to the interpersonal differences between boys and girls in peer effectiveness. In a prospective cohort study, Field et al. evaluated the peer, parent, and media influences on the formation of weight concerns and frequent dieting among preadolescent and adolescent girls and boys [44].

The proper planning for controlling childhood obesity is of great importance because this health problem not only causes physical and mental illnesses in children and adolescents but also can increase the risk of developing chronic illnesses in adulthood. Therefore, people who deal with the health and well-being of children and adolescents should use different ways to encourage families to improve their lifestyles and improve the physical health of their children.

## Study strengths and limitations

The most important strengths of this study were the use of intervention and control groups, the application of the ADDIE design model, collaboration with experts, as well as the use of FGD to select the appropriate implementation method. Another strength of this study was the use of up- to-date educational materials. However, one of the inherent disadvantages of Internet-based interventions is the lack of face-to-face contact with a trained therapist. Accordingly, it seems that the applied methodology alone was not an efficient tool for weight loss. More face-to-face training sessions could provide a possibility to track the weight loss of children, as well as separate educational feedback to reduce or gain the weight of each child to their parents. Another study limitation was the lack of direct supervision over the recommendations and training supported to schoolchildren due to home-based programs. Besides, short study duration may underestimate the intervention effect on the reduction of childhood obesity and overweight rate. Finally, the control group was not examined in the third month due to school executive restrictions on student access. Probably a comparative study in the third month could yield valuable results.

## Conclusions

In this study, an Internet-based educational intervention was used to reduce childhood obesity in Iranian society. Both the intervention and control groups gained weight after 6 months, but weight gain in the intervention group was significantly lower than the control group. Increasing the BMI was higher for boys allocated in the intervention group. Even though the weight gain also was meaningfully more moderate in the experimental group with Internet-based intervention, the six-month intervention alone is not a suitable method for weight loss in Iranian children aged 6–12 years. In addition to the Internet, it is recommended to educate the obesity-causing factors, their complications, and preventive measures by schools, in particular, teachers and student groups in a regular, continuous schedule. Furthermore, to continue learning and promote a healthy lifestyle, programs need to be developed in greater detail in terms of physical activity promotion programs, improved nutrition, and stress management with the participation of family members as well as through mass media tailored to the needs of adolescents. It is also suggested that the duration of the project be longer to make the training more effective.

## Data Availability

The datasets of the current study are available from the corresponding author on reasonable request.

## Abbreviations

BMI: Body mass index
CDC: Centers for Disease Control and Prevention
ADDIE: Analysis, design, development, implementation and evaluation
HH: Healthy habit
HEP: Healthy eating plate
RNP: Regular nutritional program
